# Incorporating Faculty and Student Co-leadership in Workgroup Structures

**DOI:** 10.1007/s40670-024-02129-2

**Published:** 2024-08-02

**Authors:** Chitra Kumar, D. J. Lowrie, Tracy Pritchard, Lisa Kelly

**Affiliations:** 1 College of Medicine, University of Cincinnati, Cincinnati, OH USA; 2 Department of Medical Education, College of Medicine, University of Cincinnati, Cincinnati, OH USA; 3 Office of Medical Education, College of Medicine, University of Cincinnati, Cincinnati, OH USA; 4 Department of Ophthalmology, University of Cincinnati College of Medicine, 231 Albert Sabin Way, 5th Floor, Cincinnati, OH 45267-0527 USA

**Keywords:** Leadership, Co-leadership, Medical education curriculum, Student-led initiatives

## Abstract

In 2020, the Dean of the University of Cincinnati College of Medicine created a college-wide task force to address inequities in the medical school curriculum. The task force consisted of four workgroups, each co-chaired by a faculty member and a student. This co-leadership model was chosen because it provided diverse perspectives and addressed the typical faculty-student power differentials. This co-leadership model balanced knowledge of medical education curriculum with the student experience. It also provided the opportunity for the co-chairs to take on new roles in leadership development: the student assumed a leadership role running the workgroup with guidance from the faculty member, while the faculty member gained insight and perspective on diversity and inclusion from the student. The purpose of this article is to describe our lessons learned during this co-leadership process.

## Introduction

With increased national attention on senseless acts of discrimination, hate, and racism across the USA, most medical schools have renewed efforts to reexamine their curricula for disparities and bias [1]. These efforts generally include building awareness with discussions around race and gender within the context of medicine, along with increasing faculty training. Although some of these efforts are initiated by the administration or faculty, many are student-driven [2, 3]. This corresponds to a general trend where students are successfully taking on prominent roles in initiatives in medical education, especially in the areas of wellness, public health emergencies, diversity, inclusion, and the transition to residency [4].

As students take on more leadership roles, collaborative efforts between faculty and students are becoming increasingly commonplace [5, 6]. Student involvement in medical education is particularly fruitful as medical students are highly motivated and can provide a unique perspective often not apparent to medical educators [7, 8]. Moreover, faculty working collaboratively with students allows for a better learning environment as students help the College of Medicine modify the curriculum and extracurricular activities to stay up to date with the evolving culture of medicine [7, 8]. Medical students are eager and interested in engaging in the transformation and innovation of medical education; however, their voices are underrepresented in the medical education scholarship [9]. In a commentary from two medical student leaders who summarized student-led initiatives in medical education, they concluded that “…we should move beyond simple engagement and instead empower trainees to lead and co-create initiatives that can ultimately benefit the entire medical education community” [4].

Co-leadership models have been used in a variety of ways in healthcare, social services, training, and education [10, 11]. Despite co-leadership being used in education sectors, literature is sparse on co-leadership models involving faculty and students. Fall and Menendez (2002) presented advantages to co-leadership which include learning different ways to navigate an issue, role modeling of behaviors, and emotional safety [10]. For co-leadership models to succeed, potential pitfalls to be mindful of include negative interactions between the co-leaders (e.g., competition), conflict resolution, and delineation of responsibilities [10]. As institutions include more student engagement into curricular and pedagogical approaches, it is important to consider student-faculty partnerships and outline the benefits and strategies for successful co-leadership relationships. Some studies have shown positive outcomes of co-leadership such as improving decision-making, increasing gender parity [12], innovation, and organizational outcomes [10].

In this article, we describe how a co-leadership dyad involving a faculty member and medical student was used to advance inclusion in medical education. In this model, of co-leadership, a faculty member and a medical student work together as equal co-chairs. This model was chosen because it brought together faculty and students working toward a common goal, with each co-chair bringing to the table a unique set of skills and perspectives. These diverse skills allowed the co-chairs to benefit and learn from each other. Here, we detail the structure and logistics of this co-leadership model and present not only the advantages but also the lessons learned from this faculty-student co-leadership model.

## Structure of the Advancing Inclusion in Medical Education (AIME) Taskforce

At the request of the Dean of the University of Cincinnati College of Medicine (UCCOM), the Advancing Inclusion in Medical Education (AIME) Taskforce was created to address diversity, equity, and inclusion in our medical education curriculum and learning environment. Although the initial call was prompted by the death of George Floyd and the subsequent national call to address racial inequities, AIME was tasked to address inequities inclusive of all groups marginalized and historically underrepresented in medicine.

The approach of the AIME task force was grounded in a multidirectional leadership model [13] from its emphasis on open membership, inclusion of a co-leadership model, and empowerment of members to drive the priorities of the task force in a systematic way while preserving the autonomy of the design and process each workgroup wanted to incorporate. This structure allowed the co-leaders to pave the workgroup’s direction in a collaborative effort to reach the requested deliverables. AIME was co-chaired by an MD faculty member and a PhD staff member involved in medical education. From the taskforce of 39 members, four workgroups were formed (Preclinical Curriculum; Clinical Curriculum; Academic Success and Personal and Professional Development; and Metrics, Policies, and Procedures). Each workgroup was appointed a student and faculty co-chair. The intent of this structure was to ensure that all voices were considered as we critically reviewed our curriculum and learning experience. From our involvement in AIME, we noted that faculty brought to the table experience in leadership along with knowledge of the curriculum, while students typically had more coursework or personal experiences related to diversity, equity, and inclusion. Because of this, we found a co-leadership model to be beneficial and allow the student and faculty co-chairs to work together on equal footing. Moreover, as the task force was longitudinal, it allowed the faculty member to observe the student as they developed as a leader, incorporate feedback, and take on more responsibilities within the workgroup.

The two principal authors (CK and DJL) were selected to co-lead the Preclinical Curriculum workgroup. The student co-chair in the Preclinical Curriculum workgroup (CK) was a first-year medical student at the time of formation of AIME. This allowed her to participate fully in the committee’s work for 2 years. Most of the other workgroups were co-chaired by a more senior student. While more senior students are more experienced, they often had scheduling challenges due to their busy clinical schedules, and eventually graduated and moved on to residency. For this reason, this article focuses on the co-leadership relationship established by the Preclinical Curriculum subcommittee.

## Content Analysis of Co-chair Reflections on Their Co-leadership Experience

Each of the co-chairs of the Preclinical workgroup provided individual reflections of their experience in the co-leadership model. Content analysis was used by the co-chair of the AIME Task Force (TP) who has experience in qualitative research methods to identify themes to this co-leadership model. The themes that emerged from this analysis were motivation, collaboration/partnership, and process.

### Motivation

Both the faculty and student were motivated to join the AIME Task Force by a drive to make a difference. The student was focused on being intentional with their time in medical school to engage in meaningful work. The faculty member saw opportunities to improve the learning environment. This drive is important as it enhances their ability to be flexible and learn to compromise with each other, knowing that they’re doing so to achieve the same goal and knowing they’re both equally invested.

Interestingly, both applied to be co-chair of a workgroup not thinking they would be selected.

Student—When I started medical school, I knew I wanted to be intentional with my time. I wanted to focus on doing work that meant more to me than just lines added to a CV for residency.

Faculty—I joined AIME because I saw places where our curriculum could do more to help underrepresented students feel more comfortable and welcome. I also joined AIME because I saw things that were being done in other institutions that were not helpful, and in fact harmful, so I wanted to ensure that we did not make the same mistakes.

### Collaboration/Partnership

The effectiveness of this co-leadership model is really driven by shared collaboration and partnership. The student had pre-conceived notions of how their role would look on the workgroup based on prior experience in research where the faculty member sets the direction while the student is assigned logistical tasks. On the other hand, the faculty member had no preset goals or ideas on how to approach the complex issues that the AIME Task Force would be identifying in their recommendations. The faculty member took a participatory approach to the workgroup—inviting the student leader’s thoughts on how to approach the workgroup meetings and recognizing their positionality and lenses they bring to the collaboration and the work of the AIME Task Force. The faculty member is an experienced educator who has led many other committees before and saw this as an opportunity for the student to lead, with the faculty member taking on more of a supportive role. This co-leadership model was approached with a willingness to share the workload as equally as possible and support the other member when schedules were heavier.

Student—I was amazed, however, once I started working with [faculty co-chair]. From our first meeting, he asked me how I thought the subgroup would best serve its purpose and how we should conduct our meetings. At the end of the first co-chair meeting, he asked me if I would like to lead our subgroup meetings.

Faculty—When we started our work, I didn’t have a plan to transition this into a co-mentorship or reverse-mentorship structure. [student co-chair] enthusiastically stepped in and started taking responsibility for the work of the committee, its organization, how it ran, etc., so it was easy for me to step back and let her take more of a role in directing the group.

Student—After that, he let me lead all the meetings while he took on tasks such as scheduling meetings, taking notes, sending follow-up emails. Again, I never asked him to do that and I offered to take it off his plate but he’d remind me that he’d already had the opportunity to lead groups before and this was instead a good learning opportunity for me.

### Process

The co-chairs of the workgroup emphasized planning, debriefing, and clear communication to achieve outcomes in their workgroup. Planning involved the co-chairs meeting prior to the meeting with the full workgroup to set an agenda and prepare to-do lists ahead of the meeting. The faculty co-chair made sure that the student would know all the individuals who would be present in the room. The student co-chair then led the meeting with the larger workgroup while the faculty co-chair listened, observed, and took notes. A debriefing meeting took place after each workgroup meeting to provide time and space for feedback and confidence building. Follow-up tasks were developed and assigned. The co-chairs also discussed any adjustments that needed to be made to facilitate the progress of the group. Lastly, the co-chairs would communicate next steps to the workgroup that incorporated feedback from the group and applied adjustments discussed during the debriefing. Importantly, the co-chairs co-signed all communications to demonstrate this shared leadership model.

Faculty—For each of our subcommittee meetings, [we] met to plan how we wanted the meeting to go, what to put on the agenda, and in which order. After each meeting, we met to de-brief, summarizing what happened at our meetings, how things could have been better, and then came up with a task-list to accomplish before our next meeting. Assigning these tasks was easy, we simply divided relatively equally in some cases, and adjusted for situations where one of us was particularly busy (“Oh, you have three exams this week (or you’re directing a course this week), then I can work on creating that table, so it’s done before our next meeting.”)

Student—Before our subgroup meeting, we made sure to meet and discuss our main agenda and to-do lists. The initial subgroup meeting definitely felt odd to me. I kept waiting for [faculty co-chair] to jump in and cut me off afraid I was saying something wrong or not doing a good enough job leading. Instead, he remained silent. He jumped in a few times to offer his opinion on the discussion amongst the group members but that’s about it. Afterwards, we debriefed, and he gave me his thoughts on things to keep in mind/way to improve along with encouragement that I was doing a good job leading. This was crucial in building my confidence.

## What We Learned from Our Experience as Student/Faculty Co-leaders

While co-chairing the Preclinical Curriculum workgroup, the principal authors (CK and DJL) identified parameters that were essential for their relationship to be fruitful for both members as co-leaders of the committee, and for the advancement of the deliverables of the committee.

**Fig. 1:**
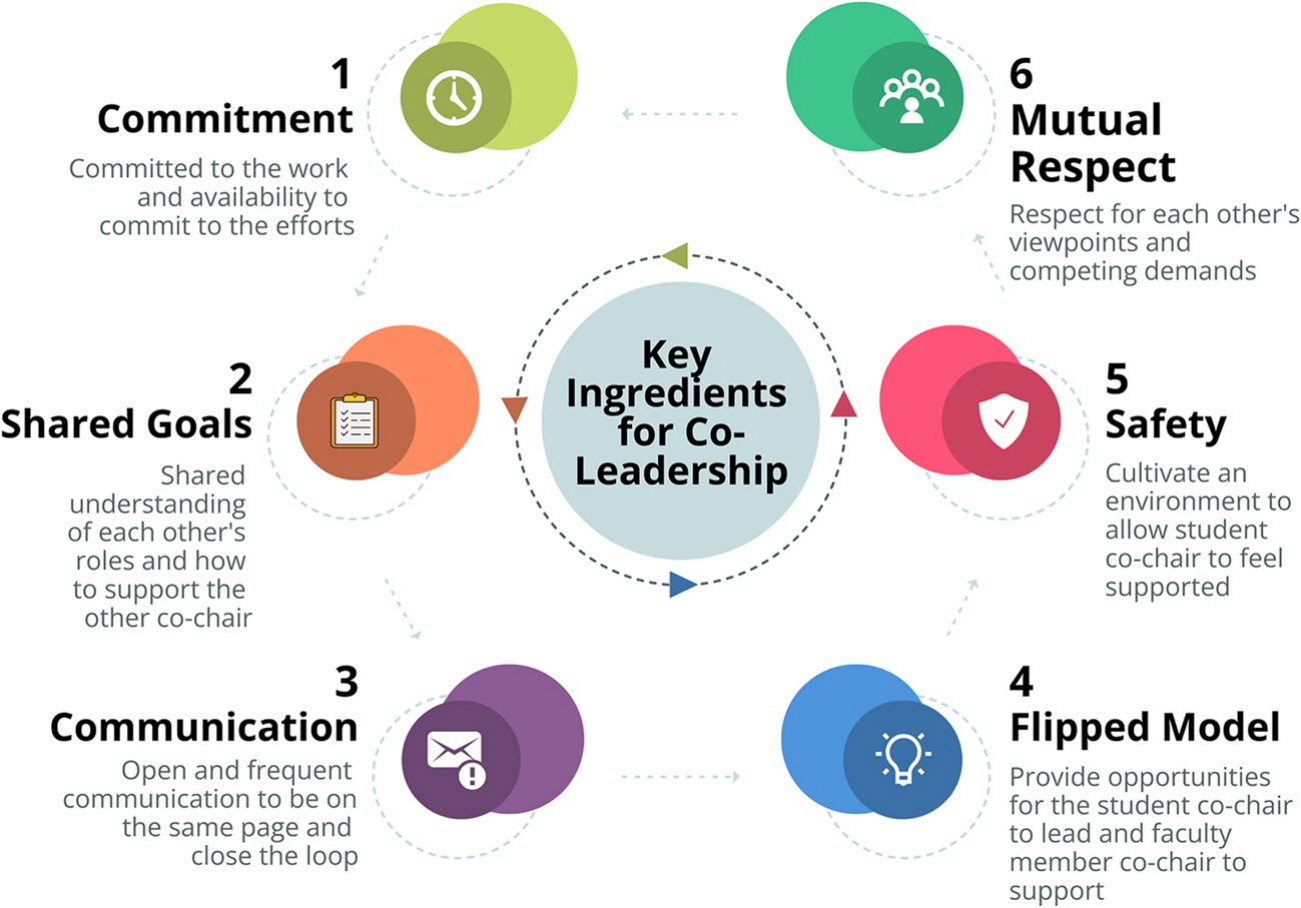
Key ingredients for co-leadership. Figure created using Venngage software

These experiences are listed below and summarized in Table 1 and outlined in a flow chart in Fig. 1. Some of these insights are intuitive yet nevertheless significant.

1. Both co-chairs must be passionate about the workgroup and committed to the importance of their mission. This ensures prompt completion of work for the workgroup and encourages the team to feel as if they are making a difference, thereby increasing motivation. For this to happen, both co-chairs must reflect on their current schedule and confirm they can appropriately dedicate time to their workgroup. This is necessary to ensure the teamwork is productive and allow both parties to get the most out of the relationship.
2. Open communication between the two co-chairs sets the stage for success.
  - This starts with something as minute as agreeing upon a preferred method of communication and prioritizing responding to each other to continue the momentum of the work.
  - It also entails taking the time to discuss their respective goals and ensure alignment. The co-chairs set the tone for the work accomplished by the workgroup. Hence, valuable time and energy is wasted if the agendas of the co-chairs do not align from the beginning. Moreover, misaligned missions can lead to disagreements and a strained relationship between the co-chairs as time progresses.
  - Periodically checking-in with each other to discuss and assign tasks which need to be accomplished closes the loop of communication and minimizes the chance of miscommunication.
3. Shared goals are essential.
  - In most cases, faculty, especially senior faculty, have had numerous previous opportunities to serve in leadership positions. Therefore, chairing a workgroup may not be as much of a career priority for promotion or recognition. This allows the flexibility to step back and become a mentor to the student in the process of leadership, such as organizing meeting agenda items, running meetings, and following up to achieve the most benefit from the meeting. In return, the faculty member can appreciate things from a student perspective, such as ways to identify and address disparities.
  - From the student’s perspective, co-chairing with a faculty member reduces the stress that comes with leading a group. For example, some stressors removed include the logistics of coordinating everyone’s schedule for meetings, learning and developing efficient agendas for each meeting, and attempting to outwardly seek feedback on leadership. With some of these barriers minimized or even removed, the focus can be on the task at hand, allowing for significantly more personal growth.
4. It is critical to use a “flipped” format of the standard student-faculty co-chair relationship. There is an inherent power differential that exists within most faculty-student collaborations. The faculty member generally dictates the direction of the group, and the student goes along with the decisions made by the faculty [14]. We revised this model. Before workgroup meetings, the co-chairs met to agree on agenda items and how to navigate difficult discussions. However, during the actual workgroup meetings, it was critical for the student to lead the discussion. In our case, the faculty member contributed to the content of the discussion as any other member but stayed in the background unless certain topics came up in the discussion where the faculty co-chair had more experience or insight. For this model to work and allow the student to focus on leading, the faculty member must be agreeable to taking on “supportive roles” such as keeping meeting notes or generating initial drafts of documents.
5. This flipped leadership model extended to outward-facing presentations as well. For example, when our workgroup presented to the larger AIME Taskforce, curriculum governance committee, or the entire student body, it was important to have the student co-chair deliver the presentation. As with the workgroup meetings, the co-chairs met briefly beforehand to agree upon the content of the presentation. This not only provided unique opportunities for growth, but gave a clear signal that students are significant stakeholders in the process. It also allowed the student to get feedback from the faculty member on their public speaking and communication skills.
6. Even the little details are significant. As communications sent by co-chairs were always a collaborative effort, they were signed by both. This may seem trivial, but messages signed by both co-chairs (even in situations where authorship of the content was from one co-chair) provided a clear signal to the workgroup and the larger taskforce that leadership of the workgroup was a joint effort.
7. Language and environment were also essential. The faculty member went out of their way to ensure the student felt comfortable in new and unfamiliar meeting settings. Before meetings with administrators unfamiliar to the student, the faculty member and the student would meet to discuss their goals for the meeting and the role of each person that would be present at the meeting. During a meeting, it was important for the faculty member to ask the student if they had anything to add to the conversation. This created a comfortable way for the student leader to speak up or request clarification.
8. Most importantly, mutual respect was the backbone of this faculty-student co-chair relationship. Leading a workgroup of this importance while either teaching or being a student at a medical school is not easy. This is where it becomes important for each co-chair to take initiative and understand each other’s timelines and be willing to take over some of the work if one chair had a period of increasing responsibilities outside the workgroup. This approach maintained workgroup momentum and further enhanced the co-leadership relationship.

**Table 1:** Examples of the key factors to success in a student-faculty co-director relationship

Clear communication on agenda (Shared Goals and Communication)	Meet a few times in the beginning to talk through ideas and make a list of short- and long-term goals to track progress and maintain momentum
Faculty member allowing student to take lead (Flipped Leadership Model)	The student oversees running all the workgroup meetings and making presentations regarding progress to the larger taskforce or the student body/administration Faculty and student meet before meetings to discuss salient points, and student checks-in frequently post-meetings to gain feedback on leadership
Faculty member helping or taking lead on mundane administrative tasks (Flipped Leadership Model)	To decrease the amount of administrative work for the student, the faculty member helps plan meetings, respond to emails, and organize logistics
Student takes a lead in driving the agenda of the committee (Flipped Leadership Model)	Student is more active in presenting ideas for accomplishing the tasks of the committee and explaining to the faculty co-chair the reasoning behind the direction of the committee
Presenting a united front (Collaboration and Importance of the Little Things)	Both co-chairs sign all emails, and both co-chairs are invited to attend all meetings
Developing a comfortable environment (Mentoring for Leadership; Providing Safety)	In meetings with administrators or other faculty members, it is easy for the student to feel overwhelmed. Obvious vocal support from the faculty co-chair in ways of asking for opinions or asking student to chime in allows the student to feel more empowered to speak up
Mutual respect (Communication and Mutual Respect)	Continuous appreciation and respect for each other in ways of stepping in when the student knows the faculty member has a busy week or vice versa allows the relationship to truly flourish and for both individuals to feel valued

## Conclusions

The workgroup co-chair organization of AIME inherently provided an avenue toward co-leadership and collaboration between students and faculty. As we have discovered, co-leadership between faculty and students can result in mentoring opportunities within the organization of groups involved in working on initiatives in medical education. This creates a powerful experience for both the student and faculty member, as both have something to contribute to and learn from the experience, especially in a longitudinal manner. Moreover, it provides ownership for all involved and gives natural outward projection that the resulting initiative is indeed a collaborative effort. 

## Data Availability

Additional data from the Co-Chair Reflections available on request.
